# Cerebral Microbleeds Detected Using 3.0T Magnetic Resonance Imaging in 2,003 Patients with Ischemic or Hemorrhagic Stroke

**DOI:** 10.31662/jmaj.2019-0002

**Published:** 2019-05-16

**Authors:** Tetsuya Abe, Masaki Takao, Hiroaki Kimura, Kazunori Akaji, Ban Mihara, Norio Tanahashi, Takashi Kanda

**Affiliations:** 1 Department of Neurology, Saitama Medical University International Medical Center, Hidaka, Japan; 2 Department of Neurology and Clinical Neuroscience, Yamaguchi University Graduate School of Medicine, Ube, Japan; 3 Department of Neurology, Mihara Memorial Hospital, Isesaki, Japan; 4 Department of Neurosurgery, Mihara Memorial Hospital, Isesaki, Japan

**Keywords:** cerebral microbleeds, magnetic resonance image, ischemic/hemorrhagic stroke

## Abstract

**Introduction:**

Compared with 1.5T magnetic resonance imaging (MRI), using 3.0T MRI makes it easier to detect cerebral microbleeds (CMBs). We used 3.0T MRI to investigate the backgrounds, risk factors, and number and location of CMBs in patients with ischemic or hemorrhagic stroke.

**Methods:**

We extracted data on clinical characteristics, risk factors, and number and location of CMBs in 2,003 patients treated between January 2010 and December 2014 within one week of stroke occurrence. We then carried out multivariate analysis of the data.

**Results:**

CMBs were present in 1,025 patients. The numbers of CMBs in ischemic stroke and hemorrhagic stroke patients were 9,410 and 6,419, respectively. Patients with CMBs showed significantly higher rates of cognitive impairment (p < 0.001, odds ratio [OR] = 1.514), hypertension (p < 0.001, OR = 3.145), previous history of stroke (p < 0.001, OR = 1.782), and presence of hemorrhagic stroke (p < 0.001, OR = 2.066). The use of antithrombotic medication before the stroke did not affect the incidence of CMBs. In ischemic stroke patients, patients with small vessel occlusion had a significantly greater rate of previous history of hemorrhagic stroke (p = 0.046) and number of patients with CMBs (p < 0.001) than those with cardioembolism.

**Conclusions:**

CMBs were well observed in patients with small vessel disease, and hypertension was an important factor in ischemic and hemorrhagic stroke. Antithrombotic medication is not associated with the development of CMBs if adequate antihypertensive therapy is provided.

## Introduction

Cerebral microbleeds (CMBs) occur owing to extravascular leakage of a small number of red blood cells from broken capillary vessels. The leaked red blood cells are engulfed by macrophages surrounding the vessel and accumulate in the form of hemosiderin. They are visualized as T2-weighted gradient echo (GRE) images on cranial magnetic resonance imaging (MRI) and as hypointense lesions on susceptibility-weighted images (SWI) ^(1)^. CMBs occur in cerebral amyloid angiopathy, hypertensive vascular disease, ischemic or hemorrhagic stroke, Alzheimer’s disease, cerebral autosomal dominant arteriopathy with subcortical infarction, leukoencephalopathy, and normal aging ^(2), (3)^.

Compared with 1.5T MRI, 3.0T MRI has twice the signal intensity, approximately four times the susceptibility effect, and better resolution, which enables clearer images to be acquired within a short time and enables easier detection of CMBs ^(4), (5)^.

In the present study, we used 3.0T MRI data to carry out a multivariate analysis of patient characteristics and risk factors for the number and location of CMBs in ischemic and/or hemorrhagic stroke. We also provide some histopathological results of CMBs.

## Materials and Methods

### Patient selection

The study was approved by the Ethics Committee of Mihara Memorial Hospital (Approval code: 075-01 and 075-02). The present study was carried out at the Mihara Memorial Hospital (Isesaki, Gunma, Japan). We reviewed all consecutive patients with acute stroke admitted to the hospital from January 2010 to December 2014. Of the 2,531 patients admitted during this period, only 2,003 patients met the study criteria. We analyzed the patients’ clinical characteristics and risk factors, and number and location of CMBs on MRI performed within one week of admission.

### Classification of type of stroke

Based on the Trial of ORG 10172 in Acute Stroke Treatment methodology ^(6)^, ischemic stroke cases were classified as large artery atherosclerosis (LAA), small vessel occlusion (SVO), cardioembolism (CE), other determined etiology, or undetermined etiology using the results of MRI and echocardiograms of the carotid arteries. To identify cases of CE, we evaluated the results of electrocardiograms, Holter monitoring, or 24-hour monitoring. The results of the blood examination were also evaluated to identify other stroke etiologies such as vasculitis and paraneoplastic conditions.

Intracerebral and subarachnoid hemorrhage were defined as types of hemorrhagic stroke. Intracerebral hemorrhage was further classified into subcortical hemorrhage and non-subcortical hemorrhage (in the basal ganglia, thalamus, brainstem or cerebellum). The patients were categorized as having experienced ischemic stroke or hemorrhagic stroke after a review of medical records and MRI data.

### Clinical characteristics

The following clinical characteristics were extracted from electronic medical charts and discharge summaries: recent/current cigarette smoking and alcohol consumption, presence of cognitive impairment (Mini-Mental State Examination score ≤ 22) at the subacute stage of stroke, hypertension (defined as receiving antihypertensive medication or blood pressure ≥ 140/90 mmHg in repeated measurements after admission), diabetes mellitus (defined as receiving antidiabetic medication, fasting blood sugar ≥ 126 mg/dL or HbA1c ≥ 6.5%, or a casual plasma glucose ≥ 200 mg/dL), hypercholesterolemia (defined as receiving cholesterol-reducing agents, an overnight fasting cholesterol level ≥ 240 mg/dL, triglycerides ≥ 200 mg/dL, or low-density lipoprotein cholesterol ≥ 160 mg/dL), liver dysfunction (defined as a history of liver disease, receiving medication for liver disease including chronic hepatitis, or alanine transaminase ≥ 30 IU/L in repeated measurements), use of antithrombotic medication at the time of admission (antiplatelet agent or anticoagulants), previous stroke history (cerebral infarction, intracerebral hemorrhage, or subarachnoid hemorrhage), atrial fibrillation, coronary artery disease, and duration of hospitalization.

### Magnetic resonance imaging protocol

All patients underwent MRI with a 3.0T Discovery MR750W device (General Electric Healthcare, USA). T2-weighted imaging (TR/TE = 5,000/90), fluid-attenuated inversion recovery imaging (TR/TE = 10,000/80), diffusion-weighted imaging (TR/TE = 70/3,300), GRE imaging (TR/TE = 500/15), and magnetic resonance angiography were performed in all patients.

### Identification of cerebral microbleeds

CMBs were defined as hypointense lesions within the cortex measuring ≤ 5 mm in diameter on GRE images. Symmetrical, small, hypointense lesions in the globus pallidus were excluded in the present study, as they may represent calcifications or iron deposits. If cortical hypointense lesions were identified on GRE, we reviewed the lesions with T2-weighted imaging to determine whether they were intraparenchymal lesions or cortical vessels. Only intraparenchymal hypointense lesions were defined as CMBs. The locations of CMBs were classified into the hypothalamus, thalamus, putamen, caudate nucleus, internal capsule, corona radiata, midbrain, pons, medulla oblongata, middle cerebellar peduncle, cerebellar vermis, cerebellar hemisphere, and cerebellar dentate nucleus, as well as the cortical and subcortical regions of the frontal, temporal, parietal, and occipital lobes. The numbers of CMBs in each area were counted on MRI. In addition, patients who only had CMBs in the basal ganglia, brainstem, or cerebellum were assigned to the ‘deep CMBs’ group. In contrast, patients with CMBs in other areas were assigned to the ‘lobar CMBs’ group. Patients with CMBs in both areas were defined as the ‘diffuse CMBs’ group.

All images were initially reviewed and diagnosed by neurologists or neurosurgeons at the Mihara Memorial Hospital. A single neurologist (T.A.) analyzed CMBs on MRI. If the lesions were difficult to identify as CMBs, the authors (T.A. and M.T.) discussed the images and decided whether they were CMBs.

### Statistical analysis

The IBM SPSS Statistics 20 software program (IBM SPSS, Inc., Chicago, IL, USA) was used for the statistical analysis.

1) We carried out multivariate analysis regarding the clinical characteristics and risk factors of patients with CMBs versus those without.

2) Patients with CMBs were categorized into two groups according to whether they had ≥ 5 or < 5 CMBs. The presence of five or more CMBs may be associated with future risk of cerebral hemorrhage ^(7), (8)^. Multivariate analysis was performed for the clinical characteristics and risk factors of both groups.

3) These clinical characteristics were also analyzed among the three different types of ischemic stroke (LAA, SVO, and CE) using the χ^2^ test and the Student’s *t*-test. Post hoc analysis was carried out using the Tukey method.

4) The numbers of CMBs in each anatomical area were summarized in bar charts based on ischemic versus hemorrhagic stroke. In addition, ischemic stroke was divided into three types (LAA, SVO, and CE). Hemorrhagic stroke was also divided into the following three types: subcortical, non-subcortical, and subarachnoid hemorrhage.

5) Multivariate analysis of clinical characteristics and risk factors was carried out among the lobar, deep, and diffuse CMBs groups.

The odds ratios (ORs) and 95% confidence intervals were determined in each statistical analysis. A p-value < 0.05 was considered statistically significant．

### Neuropathology

Of the 2,003 patients, autopsy was performed in 16 patients; this included nine ischemic stroke patients (four with LAA, two with SVO, three with CE, and one with other determined etiology), and seven hemorrhagic stroke patients (three with subcortical hemorrhage, one with non-subcortical hemorrhage, and two with subarachnoid hemorrhage). To compare the magnetic resonance images from these 16 patients with the pathological findings, the histologic sections were prepared and dissected at the same level as the CMBs on MRI. Sections were stained using HE and Berlin blue stains. Amyloid-β (11–28) immunohistochemistry was also performed.

## Results

### Classification of the type of stroke

Of the 2,003 patients, 1,551 (77.4%) had ischemic stroke; of these, 546 (35.2%) had LAA; 490 (31.6%) had SVO, 365 (23.5%) had CE, 94 (6.1%) had other determined etiology, and 56 (3.6%) had undetermined etiology. The number of hemorrhagic stroke patients was 452 (22.6%); of these, 370 (81.9%) had intracerebral hemorrhage, and 82 (18.1%) had subarachnoid hemorrhage. Among the patients with intracerebral hemorrhage, 96 had subcortical hemorrhage and 274 had non-subcortical hemorrhage.

### Clinical characteristics

The clinical characteristics and risk factors of– all 2,003 patients are summarized in Table 1. There was no sex difference observed in the present study.

**Table 1.** Multivariate Analysis between Patients with and without Cerebral Microbleeds (CMBs).

	Total (n = 2003)	CMBs + (n = 1025)	CMBs − (n = 978)	p Value	OR
Age (mean ± SD)	71.8 ± 12.4	73.3 ± 11.6	70.3 ± 13.0	< 0.001	1.025
Male sex	1192 (60%)	612 (60%)	580 (59%)	0.022	1.017
Alcohol	564 (28%)	273 (27%)	291 (30%)	0.256	0.874
Smoking	403 (20%)	194 (19%)	209 (21%)	0.687	1.055
Cognitive Impairment	637 (32%)	387 (38%)	250 (26%)	< 0.001	1.514
Hypertension	1340 (67%)	813 (79%)	527 (54%)	< 0.001	3.145
Diabetes mellitus	531 (27%)	258 (25%)	273 (28%)	0.881	0.983
Hypercholesterolemia	403 (20%)	188 (18%)	215 (22%)	0.455	0.913
Liver dysfunction	287 (14%)	152 (15%)	135 (14%)	0.156	1.222
Antithrombotic medication before stroke	543 (27%)	233 (23%)	180 (18%)	0.646	1.073
Previous history of stroke	539 (27%)	273 (27%)	159 (16%)	< 0.001	1.782
Atrial fibrillation	403 (20%)	168 (16%)	235 (24%)	0.001	0.654
Coronary artery disease	135 (7%)	60 (6%)	75 (8%)	0.050	0.673
Length of hospital stay (mean ± SD)	12.6 ± 8.2	12.5 ± 8.3	12.8 ± 8.0	0.063	0.988
Hemorrhagic stroke	452 (23%)	288 (28%)	164 (17%)	< 0.001	2.066

OR: odds ratio, SD: standard deviation

### Identification of cerebral microbleeds

**Figure 1.**
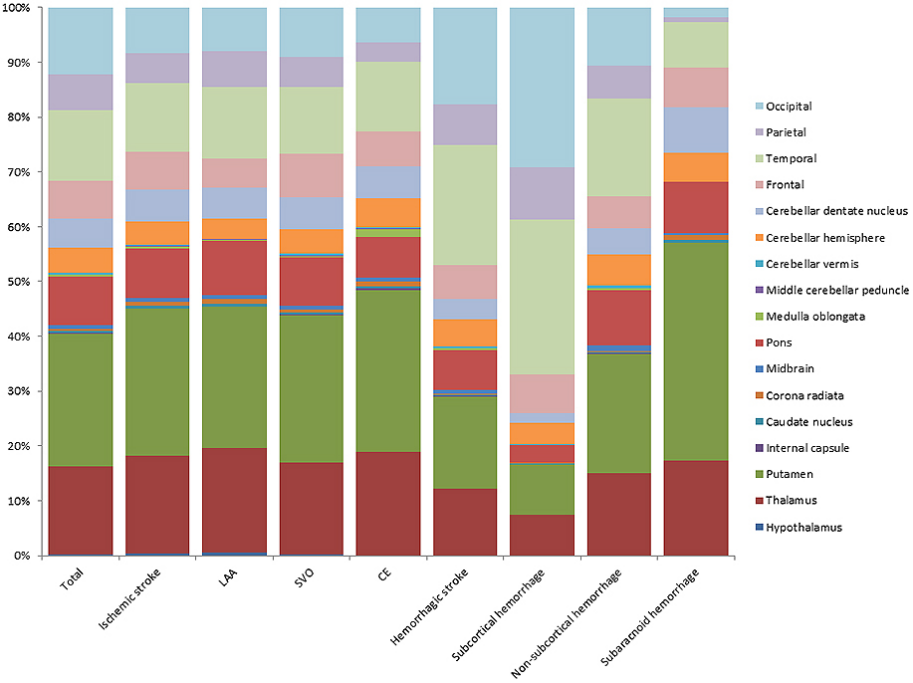
Number of CMBs in each anatomical area. CMBs were common in the putamen, thalamus, and pons, as well as in the cerebral cortex. There were generally more CMBs in the temporal cortex than in other cortices. CMBs: cerebral microbleeds, LAA: large artery atherosclerosis, SVO: small vessel occlusion, CE: cardioembolism.

CMBs were present in 1,025 patients (51.2%). Each CMB was identified strictly according to the protocol. In the 1,025 patients, we identified 15,264 CMBs. The respective numbers of CMBs in ischemic stroke and hemorrhagic stroke patients were 9,410 and 6,419. The locations of CMBs are shown in Figure 1. The numbers of CMBs in each anatomical area were 33 (0.2%) in the hypothalamus, 2,457 (16.1%) in the thalamus, 3,684 (24.1%) in the putamen, 57 (0.4%) in the caudate nucleus, 16 (0.1%) in the internal capsule, 77 (0.5%) in the corona radiata, 106 (0.7%) in the midbrain, 1,335 (8.7%) in the pons, 49 (0.3%) in the medulla oblongata, 17 (0.1%) in the middle cerebellar peduncle, 37 (0.2%) in the cerebellar vermis, 711 (4.7%) in the cerebellar hemisphere, 796 (5.2%) in the cerebellar dentate nucleus, 1,053 (6.9%) in the frontal lobes, 1,987 (13.0%) in the temporal lobes, 973 (6.4%) in the parietal lobes, and 1,876 (12.4%) in the occipital lobes.

### Multivariate analysis of patients with and without cerebral microbleeds (Table 1)

Patients with CMBs were significantly older than those without. The incidence of males was greater in patients with CMBs than in those without (p = 0.022, OR = 1.017). Patients with CMBs showed significantly higher incidences of cognitive impairment (p < 0.001, OR = 1.514), hypertension (p < 0.001, OR = 3.145), previous history of stroke (p < 0.001, OR = 1.782), and presence of hemorrhagic stroke (p < 0.001, OR = 2.066). In contrast, the patients with CMBs had a significantly lower incidence of coronary artery disease (p < 0.001, OR = 0.050) and atrial fibrillation (p < 0.001, OR = 0.654) than those without.

### Multivariate analysis of patients with five or more cerebral microbleeds versus those with less than five cerebral microbleeds (Table 2)

There were 558 patients (28%) with ≥ 5 CMBs and 1,445 (72%) with < 5 CMBs. The patients with ≥ 5 CMBs were older than those without CMBs. Compared with patients with < 5 CMBs, patients with ≥ 5 CMBs showed significantly higher incidences of cognitive impairment (p < 0.001, OR = 1.793), hypertension (p < 0.001, OR = 5.487), previous history of stroke (p < 0.001, OR = 2.166), and hemorrhagic stroke (p < 0.001, OR = 2.424). Patients with ≥ 5 CMBs had a lower incidence of atrial fibrillation (p < 0.001, OR = 0.567) than those with < 5 CMBs.

**Table 2.** Multivariate Analysis between Patients with ≥ 5 and < 5 Cerebral Microbleeds (CMBs).

	CMBs ≥ 5 (n = 558)	CMBs < 5 (n = 1445)	p Value	OR
Age (mean ± SD)	73.7 ± 11.3	71.1 ± 12.8	< 0.001	1.016
Male sex	330 (59%)	862 (60%)	0.130	1.208
Alcohol	148 (27%)	416 (29%)	0.541	0.920
Smoking	102 (18%)	301 (21%)	0.893	1.021
Cognitive Impairment	238 (43%)	399 (28%)	< 0.001	1.793
Hypertension	496 (89%)	844 (58%)	< 0.001	5.487
Diabetes mellitus	132 (24%)	399 (28%)	0.528	0.921
Hypercholesterolemia	106 (19%)	297 (21%)	0.496	1.100
Liver dysfunction	83 (15%)	204 (14%)	0.314	1.174
Antithrombotic medication before stroke	138 (25%)	275 (19%)	0.622	1.086
Previous history of stroke	175 (31%)	257 (18%)	< 0.001	2.166
Atrial fibrillation	72 (13%)	331 (23%)	< 0.001	0.567
Coronary artery disease	31 (6%)	104 (7%)	0.088	0.664
Length of hospital stay (mean ± SD)	12.2 ± 13.7	12.8 ± 8.6	0.173	0.982
Hemorrhagic stroke	187 (34%)	265 (18%)	< 0.001	2.424

OR: odds ratio, SD: standard deviation

### Analysis of the three different types of ischemic stroke (Table 3)

In comparison with patients with LAA and SVO, those with CE were older (p = 0.007 and p < 0.001, respectively) and showed significantly higher incidences of liver dysfunction (p = 0.008 and p = 0.004), antithrombotic medication before stroke (p < 0.001 and p < 0.001), and atrial fibrillation (p < 0.001 and p < 0.001), respectively. In contrast, compared with those with LAA and SVO, patients with CE had a lower incidence of smoking (p = 0.003 and p = 0.003), hypertension (p < 0.001 and p < 0.001), diabetes mellitus (p < 0.001 and p < 0.001), hypercholesterolemia (p < 0.001 and p = 0.013), and number of CMBs (p < 0.001 and p < 0.001), respectively. Patients with CE had a lower incidence of previous history of hemorrhagic stroke (p = 0.046) and number of patients with CMBs (p < 0.001) than those with SVO. In contrast, compared with those with SVO, patients with CE had a significantly higher incidence of cognitive impairment (p = 0.018). Compared with patients with LAA and CE, those with SVO had a lower incidence of coronary artery disease (p = 0.006 and p < 0.001) and duration of hospital stay (p < 0.001 and p < 0.001), respectively.

**Table 3.** Analysis among the Three Different Types of Ischemic Stroke.

	LAA (n = 546)	SVO (n = 490)	CE (n = 365)	p Value
Age (mean ± SD)	73.8 ± 11.0†	71.7 ± 11.6	76.2 ± 10.6*	< 0.001
Male sex	336 (62%)	298 (61%)	220 (60%)	0.918
Alcohol	148 (27%)	146 (32%)	103 (28%)	0.630
Smoking	125 (23%)	113 (23%)	51 (14%)*	< 0.001
Cognitive Impairment	191 (35%)	142 (29%)	138 (38%)‡	0.018
Hypertension	367 (67%)	358 (73%)	185 (51%)*	< 0.001
Diabetes mellitus	202 (37%)	164 (33%)	66 (18%)*	< 0.001
Hypercholesterolemia	136 (25%)	109 (22%)	52 (14%)*	< 0.001
Liver dysfunction	57 (10%)	48 (10%)	62 (17%)*	0.002
Antithrombotic medication before stroke	113 (21%)	90 (18%)	137 (38%)*	< 0.001
Antiplatelet agent	101 (18%)	82 (17%)	71 (19%)	
Anticoagulants	14 (3%)	10 (2%)	74 (20%)	
Previous history of ischemic stroke	109 (20%)	93 (19%)	91 (25%)	0.083
Previous history of hemorrhagic stroke	26 (5%)	23 (5%)	6 (2%)‡	0.033
Atrial fibrillation	31 (6%)	22 (4%)	324 (89%)*	< 0.001
Coronary artery disease	47 (9%)	17 (3%)§	46 (13%)	< 0.001
Length of hospital stay (mean ± SD)	12.0 ± 6.7	9.8 ± 4.4§	13.3 ± 7.5	< 0.001
CMBs presence	268 (49%)	282 (58%)	142 (39%)‡	< 0.001
Antithrombotic medication	68/268 (25%)	58/282 (21%)	58/142 (41%)	
Number of CMBs	3,280	4,602	1,213*	< 0.001

*CE vs. LAA and SVO, †LAA vs. SVO, ‡SVO vs. CE, §SVO vs. LAA and CE. CMBs: cerebral microbleeds, LAA: large artery atherosclerosis, SVO: small vessel Cocclusion, CE: cardioembolism, SD: standard deviation

### Number of cerebral microbleeds in each anatomical area (Figure 1)

In patients with ischemic stroke, we excluded ‘other determined etiology’ (n = 94) and ‘other undetermined etiology’ (n = 56) from subtype analysis owing to the small numbers of patients in these groups (Figure 1). Therefore, the analysis of ischemic stroke subtypes was only carried out in patients with LAA (n = 546, CMBs = 3,280), SVO (n = 490, CMBs = 4,602), and CE (n = 365, CMBs = 1,213). In all hemorrhagic stroke patients (n = 370, CMBs = 6,193), there were 96 with subcortical hemorrhage (CMBs = 2,377), 274 with non-subcortical hemorrhage (CMBs = 3,816), and 82 with subarachnoid hemorrhage (CMBs = 226). In patients with ischemic stroke, CMBs were most frequently observed in the putamen, followed by the thalamus and temporal lobe.

For all intracerebral hemorrhages, CMBs were most frequently seen in the temporal lobe, followed by the occipital lobe and putamen. In patients with subcortical hemorrhage, CMBs were most common in the occipital, temporal, and parietal lobes. For non-subcortical hemorrhages, CMBs were most commonly seen in the putamen, followed by the temporal lobe and thalamus. In patients with subarachnoid hemorrhage, CMBs were most frequently observed in the putamen, followed by the thalamus and pons (Figure 1).

### Multivariate analysis of clinical backgrounds and risk factors (Table 4)

In 1,025 patients with CMBs, there were 100 patients with lobar CMBs (33 [33%] with LAA, 16 [16%] with SVO, 21 [21%] with CE, seven [7%] with undetermined etiology, 12 [12%] with subcortical hemorrhage, seven [7%] with non-subcortical hemorrhage, and four [4%] with subarachnoid hemorrhage), 466 patients with deep CMBs (120 [26%] with LAA, 143 [31%] with SVO, 62 [13%] with CE, four [1%] with other determined etiology, 19 [4%] with undetermined etiology, 13 [3%] with subcortical hemorrhage, 88 [19%] with non-subcortical hemorrhage, and 17 [4%] with subarachnoid hemorrhage), and 459 patients with diffuse CMBs (115 [25%] with LAA, 124 [27%] with SVO, 58 [13%] with CE, three [1%] with other determined etiology, 12 [3%] with undetermined etiology, 38 [8%] with subcortical hemorrhage, 100 [22%] with non-subcortical hemorrhage, and nine [2%] with subarachnoid hemorrhage).

**Table 4.** Factors Associated with the Presence of Lobar or Deep Cerebral Microbleeds (CMBs).

	Lobar (n = 100)	Deep (n = 466)	Diffuse (n = 459)	p Value
Age (mean ± SD)	75.7 ± 12.0	71.8 ± 12.4*	74.2 ± 10.5	0.001
Male sex	60 (60%)	290 (62%)	262 (57%)	0.297
Alcohol	22 (22%)	144 (31%)†	107 (23%)	0.019
Smoking	16 (16%)	107 (23%)†	71 (15%)	0.011
Cognitive Impairment	41 (41%)	128 (27%)*	218 (47%)	< 0.001
Hypertension	65 (65%)	346 (74%)	401 (87%)‡	< 0.001
Diabetes mellitus	28 (28%)	120 (26%)	111 (24%)	0.661
Hypercholesterolemia	12 (12%)	87 (19%)	89 (19%)	0.215
Liver dysfunction	14 (14%)	81 (17%)	57 (12%)	0.105
Antithrombotic medication before stroke	33 (33%)	115 (25%)	144 (31%)	0.147
Previous history of stroke	25 (25%)	117 (25%)	216 (47%)‡	< 0.001
Atrial fibrillation	24 (24%)	75 (16%)	68 (15%)	0.079
Coronary artery disease	8 (8%)	26 (6%)	26 (6%)	0.631
Length of hospital stay (mean ± SD)	13.7 ± 11.1	12.5 ± 9.2	12.1 ± 6.2	0.247
Number of CMBs	860	2,057	12,932‡	< 0.001

*deep vs. lobar and diffuse, †deep vs. diffuse, ‡lobar and deep vs. diffuse SD: standard deviation

Patients with deep CMBs were younger than patients with lobar and diffuse CMBs (p = 0.007 and p = 0.004, respectively). Furthermore, patients with deep CMBs had a greater incidence of alcohol drinking (p = 0.026) and smoking (p < 0.001) than patients with diffuse CMBs. Compared with patients with lobar and deep CMBs, patients with diffuse CMBs had a greater incidence of hypertension (p < 0.001 and p < 0.001), previous history of stroke (p = 0.005 and p < 0.001), and number of CMBs (p < 0.001 and p < 0.001), respectively.

### Neuropathology

**Figure 2.**
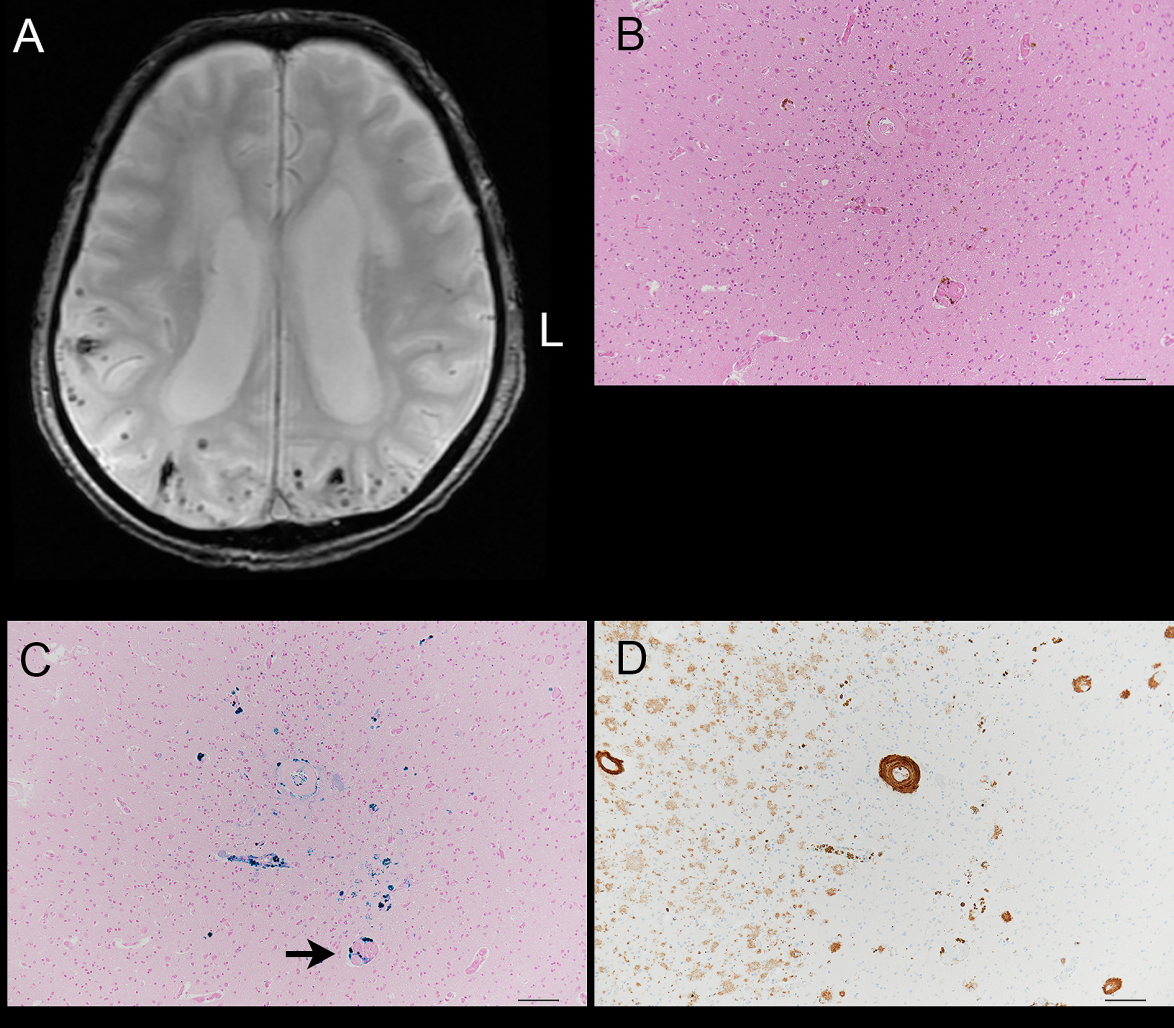
Images from a 90-year-old male with cerebral subcortical hemorrhage. A. T2-weighted gradient echo images on cranial magnetic resonance imaging. Cerebral microbleeds (CMBs) can be seen in the temporal and occipital cortices. B. Hematoxylin and eosin stain. Deposits of hemosiderin can be seen in the occipital lobe cortex. The accumulation of the hemosiderin deposits probably correspond to one CMB. Bar = 100 µm. C. Berlin blue stain. Deposits of iron are present in the perivascular regions (arrow). Bar = 100 µm. D. The vascular wall within the CMB was immunoreactive to monoclonal antibody raised against amyloid-β (11–28). Amyloid-β immunoreactive diffuse plaques are also present. Bar = 100 µm.

Although we tried to obtain additional sections from the wet preserved tissues, including CMBs on MRI, there were only two patients in whom deposition of hemosiderin was identified at the level corresponding to the position of the CMBs on MRI. One case was an LAA patient in whom hemosiderin was present in the left thalamus; the surrounding vascular walls had severe arteriosclerotic change and seemed fragile. The second case had cerebral subcortical hemorrhage; hemosiderin was seen around the blood vessels immunoreactive to amyloid-β antibody in the occipital cortex (Figure 2).

## Discussion

Although a large number of CMBs were previously reported in patients using 1.5T MRI ^(2)^, our study is the first analysis of a large number of patients using only 3.0T MRI. Our major findings were the following: 1) CMBs were particularly associated with hypertension; 2) the use of antithrombotic medication before the stroke did not affect the presence of CMBs; and 3) CMBs in the temporal lobe were common in patients with hypertensive changes.

### Classification of type of stroke and identification of cerebral microbleeds

Our cohort had a similar distribution pattern of stroke types as reported in a previous epidemiological analysis ^(9)^. Therefore, the results of our study may apply to clinical practice in Japan.

Previous studies using 1.5T MRI showed that CMBs were present in approximately 18%–39% of cases ^(10), (11), (12)^. In contrast, we found that more than 50% of the patients had CMBs in the central nervous system. We also found a relatively high incidence of diffuse CMBs (deep + lobar) in comparison with previous studies using 1.5T MRI. CMBs in the cerebral cortex may be more easily detected with 3.0T MRI. Therefore, it was difficult to compare our results to those of previous data obtained by 1.5T MRI. Although we have no 1.5T MRI data in the cohort, our results suggest that CMBs need to be analyzed using 3.0T MRI for research purposes.

On the other hand, the CMBs positive rate of SWI sequence was higher than that of GRE images ^(5), (13)^. In the present study, however, SWI was carried out in a limited number of cases. We cannot conclude the usefulness of SWI in our cohort.

### Cerebral microbleeds were particularly associated with hypertension

Hypertension, hemorrhagic stroke, and previous stroke history may be common risk factors for the development of CMBs in most patients^(14)^. Cognitive impairment is well known to be associated with CMBs, as was found in the present results. Although the rate of atrial fibrillation was low in patients with CMBs, it is difficult to conclude why atrial fibrillation may reduce the development of CMBs.

### The use of antithrombotic medication before the stroke did not affect the presence of cerebral microbleeds

The use of antithrombotic medication before the stroke did not affect the incidence of CMBs. In fact, the association between antithrombotic medication and CMBs remains controversial ^(15), (16), (17)^. A study reported that adequate antihypertensive therapy may protect against the development of new CMBs ^(15)^. Because we analyzed data from patients treated after 2010, most of them probably received adequate antihypertensive therapy.

Many studies reported that patients with ≥ 5 CMBs who are taking antithrombotic medication are at higher risk of future intracerebral hemorrhage ^(7), (8)^. We found no differences in the incidence of antithrombotic medication between cases with ≥ 5 versus < 5 CMBs. In contrast, hemorrhagic stroke and hypertension were more frequent in cases with ≥ 5 CMBs. We believe that antihypertensive treatment is important to prevent the development of CMBs. In the future, we need to analyze whether the antithrombotic medications with antihypertensive medications prevent the development of CMBs.

### Cerebral microbleeds in the temporal lobe are common in patients with hypertensive changes

CMBs are reportedly frequently present in the basal ganglia, pons, and dentate nucleus of the cerebellum ^(18)^. Our pathologic analysis showed thickening of the small vessel walls surrounded by hemosiderin, which is consistent with hypertensive changes (data not shown). In addition, CMBs were frequently observed in the temporal cortex and subcortical white matter. Although some CMBs in the temporal cortex may be associated with cerebral amyloid angiopathy, hypertensive small vessel disease may be also important for development of CMBs in the region. In the present study, the number of autopsies performed was limited. Therefore, the pathologic basis of CMBs in the temporal cortex needs to be evaluated in further.

### Analysis of the three different types of ischemic stroke

CMBs were frequently observed in patients with SVO (58%). The incidence of CMBs in SVO patients was higher than that in CE patients (Table 3). As to the treatment, the rate of antithrombotic medication usage in patients with SVO was only 21%. Similarly, the rate of antithrombotic medication usage in CE patients with CMBs was 41%. This may suggest that CE may not strongly affect the development of CMBs.

There was no difference in the presence of CMBs between LAA and SVO patients. In addition, no difference in the rates of hypertension was observed between LAA and SVO patients. Therefore, we believe that treatment of hypertension is important to prevent the development of CMBs.

### Neuropathology

Although we could only analyze a limited number of cases both neuroradiologically and neuropathologically, the results clearly showed the association between cerebral amyloid angiopathy and CMBs in the cerebral cortex. However, one study reported that amyloid-β deposits are independent of the presence of CMBs ^(19)^.

As hypertension is common in lobar CMBs patients, hypertension may also cause those cortical vessels to develop CMBs. Previous studies report that deep CMBs are associated with arteriosclerosis ^(20)^. However, we evaluated an autopsy case of CMBs in the putamen identified using post-mortem 3.0T MRI. Pathologically, the lesion was a cystic lacunar infarct with hemosiderin without definite arteriosclerosis (personal data, M.T.). Therefore, the precise pathologic backgrounds of CMBs need to be clarified in future by studies with well-designed methodology.

### Limitations of the present study

As the present study was a retrospective analysis, clinical information was only obtained from medical charts. Furthermore, we were not able to analyze patients that had not experienced stroke. Although 3.0T MRI was carried out in all patients, the angle of the images may have been slightly different among cases. Finally, the number of cases in which neuroimaging and neuropathologic correlations could be analyzed were limited.

### Conclusions

We compared the clinical characteristics and risk factors for CMBs identified by 3.0T MRI in patients with ischemic or hemorrhagic stroke. Using 3.0T MRI, CMBs were relatively common neuroradiological findings in ischemic and hemorrhagic stroke patients. Compared with previous studies, lobar CMBs were also common in many patients. Furthermore, CMBs were common in patients with small vessel disease. The data suggest that hypertension is an important factor in all types of CMBs. Our results indicate that antithrombotic medication may help prevent CMBs if adequate antihypertensive therapy is provided.

## Article Information

### Conflicts of Interest

None

### Acknowledgement

We thank Kelly Zammit, BVSc, from Edanz Group (www.edanzediting.com/ac) for editing a draft of this manuscript.

### Author Contributions

Tetsuya Abe, M.D., corresponding author. Masaki Takao, M.D., Ph.D., neuropathologic analysis, drafting manuscript. Hiroaki Kimura, M.D., Ph.D., clinical analysis. Kazunori Akaji, M.D., Ph.D., clinical analysis. Ban Mihara, M.D., Ph.D., clinical analysis. Norio Tanahashi, M.D., Ph.D., clinical analysis. Takashi Kanda, M.D., Ph.D., revising manuscript critically for important intellectual content.

### Approval by Institutional Review Board (IRB)

Approval code: 075-01 and 075-02. Institution: Mihara Memorial Hospital.

## References

[1] Yamaguchi S, Kobayashi S. Epidemiology of acute stroke in Japan: Japan Standard Stroke Registry Study. Jpn J Stroke. 2014;36(5):378-84.

[2] Poels MM, Vernooij MW, Ikram MA, et al. Prevalence and risk factors of cerebral microbleeds: an update of the Rotterdam scan study. Stroke. 2010;41(10):S103-6. 20876479 10.1161/STROKEAHA.110.595181

[3] Gregoire SM, Scheffler G, Jäger HR, et al. Strictly lobar microbleeds are associated with executive impairment in patients with ischemic stroke or transient ischemic attack. Stroke. 2013;44(5):1267-72. 23482601 10.1161/STROKEAHA.111.000245

[4] Scheid R, Ott DV, Roth H, et al. Comparative magnetic resonance imaging at 1.5 and 3 Tesla for the evaluation of traumatic microbleeds. J Neurotrauma. 2007;24(12):1811-6. 18159992 10.1089/neu.2007.0382

[5] Cheng AL, Batool S, McCreary CR, et al. Susceptibility-weighted imaging is more reliable than T2*-weighted gradient-recalled echo MRI for detecting microbleeds. Stroke. 2013;44(10):2782-6. 23920014 10.1161/STROKEAHA.113.002267

[6] Adams HP Jr, Bendixen BH, Kappelle LJ, et al. Classification of subtype of acute ischemic stroke. Definitions for use in a multicenter clinical trial. TOAST. Trial of Org 10172 in Acute Stroke Treatment. Stroke. 1993;24(1):35-41. 7678184 10.1161/01.str.24.1.35

[7] Soo YO, Yang SR, Lam WW, et al. Risk vs benefit of anti-thrombotic therapy in ischaemic stroke patients with cerebral microbleeds. J Neurol. 2008;255(11):1679-86. 19156486 10.1007/s00415-008-0967-7

[8] Gregoire SM, Jager HR, Yousry TA, et al. Brain microbleeds as a potential risk factor for antiplatelet-related intracerebral haemorrhage: hospital-based, case-control study. J Neurol Neurosurg Psychiatry. 2010;81(6):679-84. 20522874 10.1136/jnnp.2009.198994

[9] Gregg NM, Kim AE, Gurol ME, et al. Incidental cerebral microbleeds and cerebral blood flow in elderly individuals. JAMA Neurol. 2015;72(9):1021-8. 26167811 10.1001/jamaneurol.2015.1359 PMC4724412

[10] Kimura K, Aoki J, Shibazaki K, et al. New appearance of extraischemic microbleeds on T2*-weighted magnetic resonance imaging 24 hours after tissue-type plasminogen activator administration. Stroke. 2013;44(10):2776-81. 23887835 10.1161/STROKEAHA.113.001778

[11] Kakuda W, Thijs VN, Lansberg MG, et al. Clinical importance of microbleeds in patients receiving IV thrombolysis. Neurology. 2005;65(8):1175-8. 16247042 10.1212/01.wnl.0000180519.27680.0f

[12] Derex L, Nighoghossian N, Hermier M, et al. Thrombolysis for ischemic stroke in patients with old microbleeds on pretreatment MRI. Cerebrovasc Dis. 2004;17(2-3):238-41. 14718753 10.1159/000076123

[13] Viswanathan A, Chabriat H. Cerebral microhemorrhage. Stroke. 2006;37:550-5. 16397165 10.1161/01.STR.0000199847.96188.12

[14] Wardlaw JM, Lewis SC, Keir SL, et al. Cerebral microbleeds are associated with lacunar stroke defined clinically and radiologically, independently of white matter lesions. Stroke. 2006;37(10):2633-6. 16946155 10.1161/01.STR.0000240513.00579.bf

[15] Yamashiro K, Tanaka R, Okuma Y, et al. Associations of durations of antiplatelet use and vascular risk factors with the presence of cerebral microbleeds. J Stroke Cerebrovasc Dis. 2014;23(3):433-40. 23635924 10.1016/j.jstrokecerebrovasdis.2013.03.027

[16] Kim CK, Kwon HT, Kwon HM. No significant association of aspirin use with cerebral microbleeds in the asymptomatic elderly. J Neurol Sci. 2012;319(1-2):56-8. 22632777 10.1016/j.jns.2012.05.017

[17] Lovelock CE, Cordonnier C, Naka H, et al. Edinburgh Stroke Study Group. Antithrombotic drug use, cerebral microbleeds, and intracerebral hemorrhage: a systematic review of published and unpublished studies. Stroke. 2010;41(6):1222-8. 20431083 10.1161/STROKEAHA.109.572594

[18] Lee SH, Kwon SJ, Kim KS, et al. Topographical distribution of pontocerebellar microbleeds. Am J Neuroradiol. 2004;25(8):1337-41. 15466328 PMC7975477

[19] Fisher M, French S, Ji P, et al. Cerebral microbleeds in the elderly: a pathological analysis. Stroke. 2010;41(12):2782-5. 21030702 10.1161/STROKEAHA.110.593657 PMC3079284

[20] Wang Z, Soo YO, Mok VC. Cerebral microbleeds. Stroke. 2014;45:2811-7. 25028449 10.1161/STROKEAHA.114.004286

